# Management of Myosin Heavy Chain 11–Associated Familial Thoracic Aortic Aneurysm and Dissection During Pregnancy in Two Siblings

**DOI:** 10.1055/a-2774-6679

**Published:** 2026-01-09

**Authors:** Anthony Talea Pohahau, Ishaq J. Wadiwala, Lidija McGrath, Amy Hermesch, Sherene Shalhub, Castigliano M. Bhamidipati, Julie W. Doberne

**Affiliations:** 1 Division of Vascular Surgery, Department of Surgery, Oregon Health and Science University, Portland, Oregon, United States; 2 Knight Cardiovascular Institute, Section of Adult Congenital Heart Disease, Oregon Health and Science University, Portland, Oregon, United States; 3 Department of Obstetrics and Gynecology, Oregon Health and Science University, Portland, Oregon, United States; 4 Division of Cardiothoracic Surgery, Department of Surgery, Oregon Health and Science University, Portland, Oregon, United States

**Keywords:** Myosin Heavy Chain 11, fTAAD, pregnancy and aortic dissection, HTAD, patent ductus arteriosus

## Abstract

Two pregnant siblings presented with thoracic aortic dissection during the second trimester. A pathogenic *MYH11* was identified following the first sibling's diagnosis. The second sibling, previously known to be at risk but lost to follow-up, reengaged during pregnancy, tested positive for the familial variant, and dissected before her initial evaluation. This case highlights the importance of genetic diagnosis, surveillance, and multidisciplinary care in managing heritable thoracic aortic disease during pregnancy.

## Introduction

Heritable thoracic aortic disease (HTAD) in the context of pregnancy compounds a potentially life-threatening condition that can lead to aortic aneurysms, dissection, or rupture with increased risk of aortic events due to physiological changes in vascular load and wall stress. Variants in genes such as *FBN1* , *TGFBR1* / *2* , and *ACTA2* are commonly linked to HTAD. 1 2 Myosin Heavy Chain variants, *MYH11* , are a less common, autosomal dominant gene associated with familial thoracic aortic aneurysm and dissection (fTAAD), often presenting without syndromic features. 1 Management of HTAD during pregnancy requires complex multidisciplinary coordination and careful timing of delivery and aortic repair. 1 Genetic diagnosis and longitudinal follow-up are critical components of care, yet gaps in delivery may occur. We present two siblings with the same *MYH11* variant who experienced thoracic aortic dissection during their second trimesters of pregnancy, highlighting the importance of preconception counseling in at-risk individuals. This case underscores the value of early genetic identification and multidisciplinary care in managing heritable aortopathy during pregnancy.

## Case Presentation

### Sibling A

**Fig. 1:**
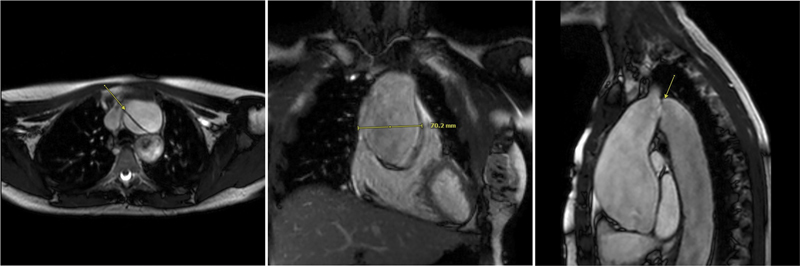
MRI images of Sibling A. Left: Axial view showing an aortic dissection flap. Middle: Coronal view demonstrating significant aortic dilation. Right: Sagittal view revealing paraductal pseudocoarctation. MRI, magnetic resonance imaging.

A 29-year-old G2P1 woman at 26 weeks of gestation was transferred from a local emergency department for management of an acute thoracoabdominal aortic dissection noted on computed tomography during evaluation for shoulder pain radiating to the abdomen. As an infant, she underwent a left thoracotomy for surgical repair of a patent ductus arteriosus (PDA). Given the patient's young age, extensive thoracoabdominal aortic dissection, history of PDA repair, and absence of hypertension and bicuspid aortic valve, HTAD was suspected. Magnetic resonance imaging (MRI) demonstrated an intimal dissection flap and aneurysmal dilation of the ascending aorta measuring 70 mm, extending from Zone 0A to Zone 0B, along with paraductal pseudocoarctation ( Fig. 1 ), and an abdominal aortic dissection involving the mesenteric branches (not shown). The patient's family history included a parent who had undergone surgical correction for PDA, a sibling with easy bruising, and a grandparent with cerebral aneurysms. Her genetic testing revealed a pathogenic MYH11 variant, c.3858 + 1G > A. At 27 weeks of gestation, the patient underwent a cesarean delivery followed immediately by valve-sparing aortic root replacement, hemiarch replacement, and repair of pseudocoarctation. Her postoperative course was uncomplicated, with regular surveillance imaging planned. The neonate was discharged from the neonatal intensive care unit (NICU) without complications.

### Sibling B

**Fig. 2:**
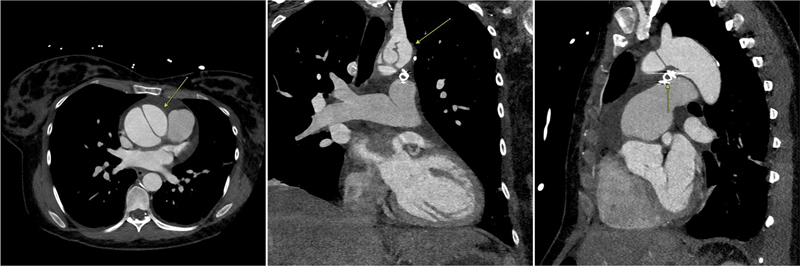
MRI images of Sibling B. Left: Axial view showing an aortic dissection flap. Middle: Coronal view demonstrating dissection extension to Zone 2. Right: Sagittal view of the ductus arteriosus with an Amplatzer Vascular Plug-2 device. MRI, magnetic resonance imaging.

**Fig. 3:**
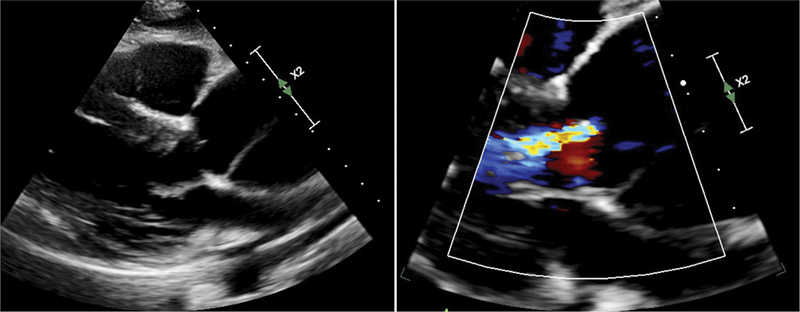
TTE images of Sibling B. Left: PLAX view with severely dilated aortic root (5.8 cm) and visible dissection flap originating at the root and extending into the ascending aorta. Right: Zoomed PLAX view highlighting mild aortic valve regurgitation, a dilated aortic root, and a dissection flap. TTE, transthoracic echocardiogram.

A 31-year-old G1P0 woman at 25 weeks of gestation was transferred from a local emergency department for evaluation of an aortic dissection identified during workup for persistent abdominal pain, nausea, anorexia, and melena. Initial examination revealed a systolic blood pressure difference > 25 mm Hg in the upper extremities. She had undergone percutaneous PDA closure with an Amplatzer Vascular Plug-II (Abbott Cardiovascular, Plymouth, MN) device nearly a decade prior to admission and was recently confirmed to carry the *MYH11* variant (c.3858 + 1G > A), diagnosed during pregnancy. Given the known family history, *MYH11* -associated fTAAD was presumed. MRI revealed a 57-mm aortic dilation at Zone 0A, with dissection extending through Zone 2, and the presence of an Amplatzer Vascular Plug-II ( Fig. 2 ). A transthoracic echocardiogram revealed severely dilated aortic root and ascending aorta, with a dissection flap originating from the aortic root and extending into the aortic arch, with mild aortic insufficiency. The left ventricular function was preserved ( Fig. 3 ). The patient endorsed intermittent abdominal pain, which was initially nonspecific, but progressed in intensity and ultimately manifested as bowel ischemia and suspected perforation. This led to fetal distress, necessitating emergency cesarean section at 26 weeks of gestation. Intraoperatively, feculent peritoneal drainage confirmed bowel perforation, requiring resection. Postoperatively, delayed abdominal closure necessitated two operative takebacks for washout, bowel resection, and salpingectomy. Sepsis and a pelvic abscess were treated expectantly. After medical optimization, the patient underwent a mechanical Bentall procedure, and patent foramen ovale closure with a stable postoperative course. Interval surveillance imaging was planned. The neonate experienced a prolonged NICU stay.

## Discussion

MYH11, an autosomal dominant gene encoding smooth muscle cell myosin, is critical for vascular stability. 3 *MYH11 is* associated with nonsyndromic HTAD, distinct from syndromic HTADs linked to *FBN1* , *TGFBR1/2* , and *ACTA2* . 1 2 We present two siblings with *MYH11* (c.3858 + 1G > A) who experienced thoracic aortic dissection during pregnancy, highlighting the challenges of managing *MYH11* -associated fTAAD. Sibling A's presentation lacked features of *FBN1* -associated Marfan syndrome or *TGFBR1/2* -associated Loeys–Dietz syndrome. Genetic testing confirmed *MYH11* and is consistent with *MYH11* -associated fTAAD. 1 4 She was counseled on the 50% inheritance risk and the need for family testing, though this was initially deferred. Sibling B's diagnosis followed Sibling A's dissection. Despite recommendations for genetic testing and surveillance, she was lost to follow-up for nearly a decade, representing a missed opportunity for early counseling and intervention. She reengaged during pregnancy, confirming the same *MYH11* variant. Unfortunately, she experienced an acute dissection before her scheduled cardio-obstetric evaluation.

For both siblings, an inpatient multidisciplinary team evaluated the risks of delayed versus combined surgical approaches. Sibling A underwent cesarean delivery followed by immediate aortic repair, whereas Sibling B required emergent delivery due to bowel perforation and fetal distress, with aortic repair delayed until her recovery. Follow-up imaging for Sibling A revealed stable dissections, whereas Sibling B's imaging showed a residual dissection confined to the brachiocephalic artery. Both neonates are thriving despite initial complications. Familial clustering of MYH11 mutations with thoracic aortic dissection and PDA underscores the importance of genetic risk assessment and counseling. 5 Systemic complications, such as cerebral arteriopathy and vascular fragility, further emphasize the need for vigilant, multidisciplinary care. 6 7 While genotype–phenotype variability in *MYH11* is recognized, its clinical implications remain uncertain. 8 Our patients, sharing the same variant but displaying phenotypic differences, contribute to understanding the complex expressivity of MYH11 mutations.

We highlight the critical importance of preconception counseling, regular surveillance, and adherence to guidelines for managing genetically mediated aortopathy. An inpatient multidisciplinary approach is essential to successfully managing evolving clinical processes and optimizing outcomes in *MYH11* -associated fTAAD during pregnancy.

A formal Institutional Review Board determination concluded that this case report does not involve human subjects research; therefore, patient consent was not required.
